# *CYP1B1* mutations in patients with primary congenital glaucoma from Saudi Arabia

**DOI:** 10.1186/s12881-014-0109-2

**Published:** 2014-09-28

**Authors:** Osama M Badeeb, Shazia Micheal, Robert K Koenekoop, Anneke I den Hollander, Manal T Hedrawi

**Affiliations:** Glaucoma unit, College of Medicine, King Abdulaziz University, Jeddah, Saudi Arabia; Departments of Ophthalmology and Human Genetics, Radboud University Medical Center, Nijmegen, The Netherlands; McGill Ocular Genetics Laboratory, Pediatric Ophthalmology unit, McGill University Health Center, Montreal, QC Canada; Pediatric Ophthalmology unit, King Fahd armed forces hospital, Jeddah, Saudi Arabia

**Keywords:** Congenital, Glaucoma, Gene, Mutation, CYP1B1

## Abstract

**Background:**

*CYP1B1* is the most commonly mutated gene in primary congenital glaucoma (PCG). This study was undertaken to identify mutations in *CYP1B1* in the Western region of Saudi Arabia.

**Methods:**

Blood of patients who had typical findings of PCG, were screened by direct sequencing of all coding exons and splice junctions of the *CYP1B1* gene.

**Results:**

34 patients were studied; 18 patients belonged to 8 families, and 16 patients were non-familial, isolated PCG. Consanguinity was found in 27/34 (79.4%) of cases. All patients were diagnosed to have bilateral PCG at birth except one child, who had glaucoma in the right eye. More males (61.8%) were affected than females (38.2%). 79.4% (27/34) of patients were solved with pathogenic mutations and 20.6% (7/34) remained unsolved. Of the solved ones, 22.2% (6/27) of patients carry a pathogenic allele on one allele while the other allele remained yet to be determined. Direct sequencing of exon 2 revealed two pathogenic variants (p.Gly61Glu, p.Glu229Lys). P.Gly61Glu substitution was found both homozygously in 63% (17/27) of cases, and heterozygously in one patient. P.Glu229Lys variant was found heterozygous in 3.7% (1/27) of cases. One pathogenic variant (p.Arg469Trp) was found in exon 3, and is present homozygously in 14.8% (4/27) of cases while four patients have this variant heterozygously. All mutations were reported previously in the Saudi population, except p.Glu229Lys. Severe cases were associated with p.Gly61Glu, and p.Arg469Trp in 50% and 30% of ten patients respectively.

**Conclusions:**

This study confirms that *CYP1B1* mutations are the most frequent cause of PCG in the Saudi population, with p.Gly61Glu being the major disease-associated mutation. P.Glu229Lys is a newly discovered mutation in our PCG patients. Patient lacking mutation in *CYP1B1* gene seems likely, to have another genetic loci involved in the pathogenesis of the disease, and need further study. Genetic studies of recessive diseases such as PCG is important in consanguineous populations, since it will increase awareness and allows genetic counseling to be offered to patients and their relatives. This will not only reduce the disease to be inherited to future generations, but will also reduce the disease burden in the community.

## Background

Primary congenital glaucoma (PCG) refers to a glaucoma occurring within the first 3 years of life, due to isolated trabeculodysgenesis [1]. It is relatively common in Saudi Arabia (SA), with an estimated incidence of 1: 2500 live births, secondary to a high prevalence of consanguinity in Saudi families [2]. It is the second most common cause of blindness in schools of the blind in SA [3]. The congenital glaucoma registry at King Khaled Eye Specialist Hospital showed that PCG geographic distributions in SA is: south region (27.8%), western province (23.6%), central region (22.2%), eastern province (11.1%), and north province (9%) [4].

The link between PCG and gene abnormalities is the initial step in the establishment of the pathophysiology of the disease. Three previous major studies [5-7] were conducted in SA, to identify the abnormal genes associated with PCG. So far *CYP1B1* was the only gene found to be associated with bilateral PCG in consanguineous Saudi Arabian families. *CYP1B1* mutations were identified in 96% of cases of the study described by Bejjani et al. [6] and in 75.9% of cases of the study described by Abu-Amero et al. [7]. Unilateral PCG were studied by Khan et al. [8] but was not associated with *CYP1B1* mutations. Expatriates were excluded from all previous gene studies [4-8].

This study was conducted to identify *CYP1B1* mutations in Saudi PCG patients with various ethnic backgrounds, and to identify those patients who carry novel mutations. Patients were collected at King Abdulaziz University Hospital, one of the major hospitals in the Western province of Saudi Arabia and a main referral centre for congenital glaucoma in the province.

## Methods

### Patients and clinical data

The study adhered to the guidelines of the Declaration of Helsinki and was approved by King Abdulaziz University Research ethics committee (Reference No. 922–12).

After signed informed consents were obtained, blood samples were collected for DNA isolation from 34 patients with PCG.

Patients were recruited from the glaucoma clinic at King Abdulaziz University Hospital (KAUH) and the pediatric ophthalmology clinic at King Fahd Armed Forces Hospital (KFAFH), in Jeddah, Kingdom of Saudi Arabia. The diagnosis of PCG was established by the following signs: (1) increased intraocular pressure (IOP) (>21 mmHg), (2) cornea diameter (>10 mm) and changes (oedema, Haab’s striae, scar), (3) optic nerve glaucomatous cupping, and by the following symptoms: photophobia, epiphora, and/or blephrospasm [1]. The medical history of all participants was taken and the family pedigrees were drawn. Patients with other ocular and systemic abnormalities were excluded.

### Molecular genetic analysis

Primers were designed for the two coding exons and splice site junctions of the *CYP1B1* gene using Primer 3 (primer sequences and PCR conditions are available on request). PCR products were visualized on 2% agarose gel and purified with PCR clean-up purification plates (NucleoFast® 96 PCR, MACHEREY-NAGEL, Germany), according to the manufacturer’s protocol. Purified PCR products were analyzed by Sanger sequencing in both directions using both forward and reverse primers in an automated DNA sequencer (Big Dye Terminator, version 3 on a 3730 DNA analyzer; Applied Biosystems, Inc., Foster City, CA). Sequencing results were aligned with the reference sequence (obtained from the hg19 human genome build) and analyzed using Vector NTI Advance (TM) 2011 software (Invitrogen). In addition, the pathogenicity of *CYP1B1* missense variants was evaluated by publically available *in silico* tools including PhyloP, Grantham, PolyPhen2, and SIFT.

### Data collection and statistical analysis

Data were collected from the medical records in a special form and then entered and stored in computer Microsoft ward. Graph Prism 5 software (GraphPad Software Inc, La Jolla, Ca USA) was used to analyze the data. Frequency distribution tables were used to present the data. Mean, standard deviation and (student’s) t-test were used to compare continuous variables.

All tests were two sided and at a 5% level of statistical significance. Fisher’s exact test also used to compare the real values of native and non-native Saudi groups.

## Results

### Demographic and medical data

Our study included 34 PCG patients: 23 patients from KAUH and 11 patients from KFAFH. 18 patients belonged to 8 families with consanguineous parents, while9 patients were unrelated, non-familial, isolated PCG cases belonging to consanguineous parents. 7 patients were unrelated, non-familial, isolated PCG belonging to non-consanguineous parents. Consanguinity was reported in 27/34 (79.4%) of cases.

All 34 patients were diagnosed to have bilateral PCG at birth except one child, who had glaucoma only in the right eye. More males (21/34) were affected than females (13/34). At presentation, the mean IOP +/− standard deviation (SD) was 30.48+/−6.93 (range 20 – 47) mmHg, the mean corneal diameter +/− SD was 12.71 +/−1.26 (range 10 – 17) mm. The mean cupping +/− SD and mean refraction +/− SD of 23 eyes with clear cornea was 0.47 +/− 0.29 (range 0.1 - 1.0), and - 4.92 +/− 5 (range +2.3 to −16) D respectively.

Twenty three cases were native Saudi, of which 21 (91%) were positive for *CYP1B1* mutations, and 2 (9%) were negative. Eleven patients were non-native Saudi, originating from Yemen (4), Sudan (3), Afghanistan (2), Burma (1), and Syria (1). Of the non-native cases, 6 (54.5%) were positive for *CYP1B1* mutations, and 5 (45.5%) were negative. Overall 27/34 (79.4%) cases carried *CYP1B1* mutations and 20.6% (7/34) patients remained unsolved. Table 1, present a comparison of demographic, medical and *CYP1B1* mutations between native and non-native Saudis. No significant differences were observed between the 2 groups, except for the mean C/D ratio, which was higher in the non-native Saudi group.

**Table 1:** **Comparison of demographic data and CYP1B1 mutations between native and non-native Saudi patients**

	**No. of patients**	**Gender (%)**		**Affected eyes**		**Cons (%)**	**Mean IOP (+/−SD) mmHg**		**Mean corneal diameter (+/−SD) mm**		**Mean C/D (+/−SD)**		**Mutation (%)**
		**M**	**F**	**R**	**L**		**R**	**L**	**R**	**L**	**R**	**L**	
Native Saudi	23	14 (60.7)	9 (39.1)	23	23	21 (91.3)	29.2 (4.9)	30.9 (6.9)	12.5 (1.3)	12.7 (1.6)	0.3 (0.3)	0.4 (0.3)	21(91)
Non-native Saudi	11	7 (63.6)	4 (36.4)	11	10	6 (54.6)	31.7 (9.07)	31.3 (9.04)	12.8 (0.7)	12.8 (0.9)	0.7 (0.2)	0.7 (0.3)	6 (54.5)
P Value		*1	*1	*1	*1	*0.4	**.302	**.887	**.481	**.849	**.0005	**.0153	**0.4

M-Male, F-Female, R-Right eye, L-Left eye, IOP-Intraocular pressure, C/D-Cup to Disc ratio, Cons = consanguinity, *Fisher’s exact test, **t-student test.

Ten patients were classified to have severe glaucoma on presentation (based on IOP level, corneal diameter and clarity) according to Al-Hazmi et al. [9] classification. 5 (50%) patients were associated with G61E, and 3 (30%) patients with R469W mutations Table 2.

**Table 2:** **Correlation between CYP1B1 mutations and Severity of PCG in native (23) and non-native (11) Saudi**

	**Severity**											
	**Mild**				**Moderate**				**Severe**			
	**None**	**G61E**	**R469W**	**E229K**	**None**	**G61E**	**R469W**	**E229K**	**None**	**G61E**	**R469W**	**E229K**
No. of Native Saudi (%)	1 (4.4)	6 (26.1)	2 (8.7)	0 (0)	0 (0)	5 (21.7)	2 (8.7)	0 (0)	1 (4.4)	4 (17.4)	2 (8.7)	0 (0)
No. of Non-Native Saudi (%)	2 (18.2)	1 (9.1)	0 (0)	0 (0)	2 (19.2)	1 (9.1)	1 (9.1)	1 (9.1)	1 (9.1)	1 (9.1)	1 (9.1)	0 (0)
Total (%)	3 (8.8)	7 (20.6)	2 (5.9)	0 (0)	2 (5.9)	6 (17.7)	3 (8.8)	1 (2.9)	2 (5.9)	5 (15.8)	3 (8.8)	0 (0)

[9] Mild: (IOP < 25 mmHg, Corneal diameter < 13 mm, Clear cornea).

Moderate: (IOP = 25-35 mmHg, corneal diameter = 13-14.5 mm, hazy cornea).

Severe: (IOP > 35 mmHg, corneal diameter > 14.5 mm, cloudy cornea).

### *CYP1B1* mutation analysis

DNA samples of all patients were screened for mutations in the *CYP1B1* gene. Pathogenic variants were detected in *CYP1B1* gene in 27 patients. 77.8% (21/27) patients were completely solved with pathogenic mutations, while 22.2% (6/27) patients were carrying a pathogenic allele on one allele while the other allele remained yet to be known. Direct sequencing of exon 2 revealed two pathogenic variants (c.182G > A: p.Gly61Glu, and c.685G > A: p.Glu229Lys). The p.Gly61Glu substitution was found homozygously in 63% (17/27) patients, of which 10 cases belong to 4 consanguineous families. The p.Glu229Lys variant was found heterozygously in 3.7% (1/27) of cases Table 3.

**Table 3:** **Mutations detected in** ***CYP1B1*** **in PCG patients from Saudi Arabia**

**Nucleotide change**	**Amino-acid change**	**Patient heterozygous**	**Patient homozygous**	**Families**	**PhyloP**	**Grantham distance**	SIFT score*	PolyPhen**	**dbSNP**
c.-1-12C > T	N/A	2	1	N/A	N/A	N/A	N/A	N/A	rs2617266
c.142C > G	p.Arg48Gly	2	2	N/A	−1.25	125	0.38	0.00	rs10012
c.355G > T	p.Ala119Ser	3	3	N/A	−0.28	99	0.79	0.00	rs1056827
c.729G > C	p.Val243Val	1	0	N/A	N/A	N/A	N/A	N/A	rs9341249
c.1328C > G	p.Ala443Gly	1	0	N/A	0.85	60	0.19	0.00	rs4986888
c.1347 T > C	p.Asp449Asp	5	3	1	N/A	N/A	N/A	N/A	rs1056837
c.182G > A	p.Gly61Glu	1	17	4	3.34	98	0.0	1.00	rs28936700
c.685G > A	p.Glu229Lys	1	0	N/A	3.6	56	0.03	0.99	Rs57865060
c.1405C > T	p.Arg469Trp	4	4	3	1.09	101	0.0	1.0	Rs28936701

[5-7,10-19] *Substitutions with a SIFT score <0.05 are predicted to be deleterious, **Polyphen scores near 1 are confidently predicted to be pathogenic. N/A = Not Applicable.

Similarly, one pathogenic variant (c.1405C > T: p.Arg469Trp) was detected in exon 3. The p.Arg469Trp was found in 29.6% (8/27) patients. The p.Arg469Trp substitution was found homozygously in four patients belonging to two consanguineous families.

The probable pathogenic mutations of the 22.2% (6/27) patients were, p.Arg469Trp in four patients, p.Gly61Glu in one patient and p.Glu229Lys in one patient, heterozygously. These six patients might have a second variant in the same gene that lies in the noncoding region i.e. intron or promoter Table 3.

In addition to those pathogenic variants, 6 other common SNPs (rs2617266, rs10012, rs1056827, rs9341249, rs4986888 and rs1056837) were also found either homozygously and heterozygously, that were not predicted to be pathogenic Table 3.

## Discussion

The populations of the Arabian Peninsula descend from a small number of founders. Their descendants established various tribes that dispersed across the kingdom. Therefore, the majority of native Saudi Arabians living today have common ancestors [5]. This is may be true in all parts of SA except the Western region, where the population consists of a different ethnic groups due to the presence of the two holy cities, Makkah and Madinah, and immigration of people from Arabic and Islamic countries to this region over the last 14 centuries. Jeddah is the sea port of the two holy cities, and thus also has a population with a high cultural and ethnical diversity. Non-native Saudi PCG patients were excluded from all previously published gene studies, and only conducted on native patients from various tribes and regions of SA [4-8]. To the best of our knowledge this is the only gene study in SA, which addresses the effect of ethnicity on gene anomalies in PCG.

The *CYP1B1* gene was selected to screen for mutations in our PCG patients, since it was considered the main candidate gene for PCG in Saudi Arabia. *CYP1B1* mutations were found in 79.4% of our patients, which is higher than that reported by Abu-Amero et al. [7] and lower than that reported by Bejjani et al. [6].

We detected *CYP1B1* mutations in 91% and 54.5% of native and non-native Saudi Arabian PCG patients, respectively. This difference in rate of mutations could be secondary to the lower consanguinity rate in the non-native Saudi population (54.6%) compared to the native population (91.3%). The rate of *CYP1B1* mutations in our non-native Saudi cases is low and comparable to populations with low consanguinity rates like India (44%) [10] and Brazil (50%) [11]. In addition, the high rate of *CYP1B1* mutations in the native Saudi PCG cases may be explained by the population structure. Since the native Saudi population descended from a limited number of founders, it is likely that one of a small number of founder mutations have arisen that explain the majority of the cases among the native Saudi populations. Two of the pathogenic variants (p.Gly61Glu, p.Arg469Trp) identified in our cases were previously reported in the literature in PCG patients of various populations (ethnic groups), including native Saudi Arabians [5-7]. The Gly61Glu mutation is the most frequent mutation in the Saudi patients of our current study as well as in patients from Kuwait. Both SA and Kuwait share a common genetic background because of the neighborhood and frequent migration routes between the two populations [12]. We identified a missense mutation (p.Glu229Lys) in one of our non-native Saudi PCG patients. This patient’s family is originally from Burma. This mutation has not been reported previously in native Saudi Arabian PCG patients [5-7], but it has been reported in Turkish [13], Indian [10], Iranian [14] patients, and in an index patient of family from Oman in heterozygous manner [15].

In a previous study of *CYP1B1* analysis by Bejjani et al. [6] in PCG patients from SA a haplotype was constructed for the six SNPs. We also constructed haplotypes ie C/C/G/G/T/A using the same SNPs which includes one intronic c.-1-12C > T and five exonic SNPs i.e. c.142C > G; R48G, c.355G > T; A119S, c.1294C > G; V432L, c.1347T > C; D449D and c.1358A > G; N453S. In their study they had observed that this distinctive haplotype is associated with the five different mutations and we also saw a same haplotype C/G/G/T/A in case of the two mutations Gly61Glu and Arg469Trp in 27 patients. This same haplotype C/C/G/G/T/A was associated with at least seven separate mutations and appears to be common in all the individuals carrying the most frequent mutation, 4340delG in CYP1B1 in Brazilian PCG patients [11,16]. However in the 7 patients which remained unsolved with *CYP1B1* in the current study, 4 patients had this haplotype T/G/T/C/C/A while three individuals were heterozygous for all these five SNPs. Bejjani et al. [6] also found that the three families unsolved with CYP1B1 mutations has a heterozygous haplotype for these five SNPs. It seems likely, that individuals lacking haplotype C/C/G/G/T/A have another genetic loci involved in the pathogenesis of PCG. We are currently exploring this possibility.

In the literature, there has not been a consistent correlation between the severity of PCG and presence and types of *CYP1B1* mutations [17]. Hollander et al. [18] found a correlation between the type of *CYP1B1* mutations and the severity of glaucoma on the basis of anterior chamber angle histological changes and difficulty in controlling IOP. Panicker et al. [19] found that severe cases (using severity index) of PCG associated with ins376A (100%), E229K (80%), E368H (72%), G61E (66.7%), and P193L (62.5%). The severity of PCG in our patients was assessed on the basis of clinical assessment of IOP level, corneal diameter, and corneal clarity at the time of presentation. We did find severe cases of PCG associated with two mutations, G61E (50%), and R469W (30%), in ten of our patients.

## Conclusions

This study confirms that *CYP1B1* mutations are the most frequent cause of PCG in the Saudi Arabian population, with p.Gly61Glu being the major disease-associated allele. The p.Glu229Lys is a new mutation in Saudi Arabian PCG patients. Genetic studies of recessive diseases such as PCG is important in consanguineous populations, since it will increase awareness and allows genetic counseling to be offered to patients and their relatives. This will not only reduce the disease to be inherited to future generations, but will also reduce the disease burden in the community.
